# Induced Hypothermia Does Not Harm Hemodynamics after Polytrauma: A Porcine Model

**DOI:** 10.1155/2015/829195

**Published:** 2015-06-11

**Authors:** Matthias Weuster, Philipp Mommsen, Roman Pfeifer, Juliane Mohr, Steffen Ruchholtz, Sascha Flohé, Matthias Fröhlich, Claudia Keibl, Andreas Seekamp, Martijn van Griensven, Ingo Witte

**Affiliations:** ^1^Department of Trauma Surgery, University of Schleswig-Holstein, 24105 Kiel, Germany; ^2^Department of Trauma Surgery, Hannover Medical School, 30625 Hannover, Germany; ^3^Department of Trauma Surgery, University of Aachen, 52074 Aachen, Germany; ^4^Department of Trauma Surgery, University of Marburg-Giessen, 35043 Marburg, Germany; ^5^Department of Trauma Surgery, University of Magdeburg, 39120 Magdeburg, Germany; ^6^Department of Trauma Surgery, University of Düsseldorf, 40225 Düsseldorf, Germany; ^7^Department of Trauma Surgery, University of Witten-Herdecke, 51109 Köln-Merheim, Germany; ^8^Ludwig Boltzmann Institute for Experimental and Clinical Traumatology, 1200 Vienna, Austria; ^9^Department of Experimental Trauma Surgery, Klinikum Rechts der Isar, Technical University Munich, 81675 Munich, Germany

## Abstract

*Background*. The deterioration of hemodynamics instantly endangers the patients' life after polytrauma. As accidental hypothermia frequently occurs in polytrauma, therapeutic hypothermia still displays an ambivalent role as the impact on the cardiopulmonary function is not yet fully understood.* Methods*. We have previously established a porcine polytrauma model including blunt chest trauma, penetrating abdominal trauma, and hemorrhagic shock. Therapeutic hypothermia (34°C) was induced for 3 hours. We documented cardiovascular parameters and basic respiratory parameters. Pigs were euthanized after 15.5 hours.* Results*. Our polytrauma porcine model displayed sufficient trauma impact. Resuscitation showed adequate restoration of hemodynamics. Induced hypothermia had neither harmful nor major positive effects on the animals' hemodynamics. Though heart rate significantly decreased and mixed venous oxygen saturation significantly increased during therapeutic hypothermia. Mean arterial blood pressure, central venous pressure, pulmonary arterial pressure, and wedge pressure showed no significant differences comparing normothermic trauma and hypothermic trauma pigs during hypothermia.* Conclusions*. Induced hypothermia after polytrauma is feasible. No major harmful effects on hemodynamics were observed. Therapeutic hypothermia revealed hints for tissue protective impact. But the chosen length for therapeutic hypothermia was too short. Nevertheless, therapeutic hypothermia might be a useful tool for intensive care after polytrauma. Future studies should extend therapeutic hypothermia.

## 1. Introduction

Therapeutic hypothermia (TH) [1] has gained increasing popularity over the last years. Widely used in elective fields of surgery [2, 3], TH takes an important role in tissue protection and facilitates a reduction of the metabolic rate [4]. Nevertheless, its positive effect is still controversially discussed as populations in cardiac arrest and traumatic brain injury studies were not quite comparable [1, 5–7]. Furthermore, hypothermia plays an ambivalent role in polytrauma patients as many suffer from accidental hypothermia (AH). AH can be distinguished by its degree Celsius (°C) as mild (33–36°C), moderate (28–32.9°C), deep (11–27.9°C), profound (6–10.9°C), and ultraprofound (<6°C) [8]. The exposure to cold environment and forced volume management induces a decrease in body core temperature. Following ATLS guidelines it is indispensable to avoid the lethal triad of accidental hypothermia, acidosis, and coagulopathy as these components aggravate patients' outcome [9]. If temperatures decline below 34°C coagulation disorders occur with reversible thrombocytopenia and thrombocytopathy. Plasmatic coagulopathy occurs below 33°C with disorders in thrombin and fibrin function [10]. The development of coagulopathy is supported under circumstances of increased heat loss, hemorrhage, and uncontrolled fluid management in trauma scenarios. Unfortunately, major complications appear in the clinical course with high mortalities correlating with hemorrhagic shock after blunt chest and penetrating abdominal trauma [11–13]. Studies have proven that hypovolemia alone is sufficient enough to cause remote organ damage potentiated by insufficient tissue oxygenation [14]. Therefore, strategies in emergency care medicine emphasize the golden hour of shock in which tissue perfusion and oxygenation need to be improved; here invasive devices for hemodynamic monitoring such as the pulmonary artery catheter have been used for goal-directed resuscitation [15]. Early detection of cardiac failure still belongs to the most challenging aspects in shock management [16]. Considering these circumstances, TH might improve organ functions after polytrauma. Superior effects of TH are still unknown.

The aim of our investigation was to analyze the hemodynamics due to therapeutic hypothermia after polytrauma and resuscitation in a porcine model. Cardiovascular parameters were documented in order to detect benefits and harmful impact of TH. We hypothesized that hemodynamics after polytrauma could benefit from mild therapeutic hypothermia. TH might be an important tool in trauma care as used in other fields of emergency care [2, 17, 18].

## 2. Methods and Material

### 2.1. Animal Care

This study was approved by the regional animal welfare Committee of Vienna, Austria, confirming the guidelines of the National Institutes of Health for the use of experimental animals. The experiment was performed in *n* = 40 male pigs (German domestic pigs, Muenichstal) aged 12 to 16 weeks and weighing 30 ± 3 kg [14, 19, 20]. Water was available ad libitum and the animals were fasted overnight. 20 pigs were assigned to a sham group with either normothermic (*n* = 10) or hypothermic conditions (*n* = 10) only receiving anesthesia and placement of arterial, venous, and urinary lines. 20 pigs were analyzed in a trauma group with blunt chest trauma, liver laceration, and hemorrhagic shock with either normothermic (*n* = 10) or hypothermic conditions (*n* = 10) after resuscitation. All experimental procedures were conducted in deep anesthesia. All animals were sacrificed 15.5 hours by bolus infusion of potassium chloride.

### 2.2. Anesthesia

The animals were kept in a supine position. Anesthesia was induced with an intramuscular application of zoletil mixture (xylazine 146 mg, ketamine 125 mg, butorphanol 25 mg, tiletamine 50 mg, and zolazepam 50 mg) in a dose rate of 1 mL/15 kg/pig. After endotracheal intubation and during preparation, anesthesia was performed using isoflurane 1.5% with rocuronium (5 mg/kg/h) and sufentanil (0.008 mg/kg/h) intravenously via ear vein access. From baseline (BL) on, anesthesia was maintained for the entire study period as total intravenous anesthesia with midazolam (0.8 mg/kg/h), rocuronium (5 mg/kg/h), and sufentanil (0.008 mg/kg/h). This medication was chosen in order to prevent any diminution in cardiopulmonary functions like a decrease of mean arterial pressure (MAP) as effectuated by inhalation anesthetics [21]. Liquids were given for all animals during the protocol per crystalloids (Ringer, 309 mosmol/L, Fresenius Kabi GmbH, Graz, Austria) in a rate of 10 mL/kg bodyweight/hour. During the whole study period, oxygenation and ventilation were continuously monitored and adapted to obtain physiologic levels with a pressure-volume-controlled setting in a ventilator (Draeger, Primus, Danvers, MA, USA). A FiO_2_ of 30%, a tidal volume of 10 mL/kg/bodyweight, 20 breaths/min, a PEEP of 3 mbar, paCO_2_ of 35–45 mmHg, and end-tidal CO_2_ of 4.5–5.5% were set.

### 2.3. Preparation of the Animals

Preparations were performed in an aseptic technique. Saline-filled catheters were placed intraluminally by preparing the following arteries and veins: left carotidal artery for arterial line (B. Braun Perfusor Line, Melsungen, Germany), left external jugular vein for drug and medical substitution by applying a 9FR-central venous catheter (Arrow Int. Reading, PA, USA), and right external jugular vein by applying a Swan-Ganz catheter for drug and medical substitution as well as measuring hemodynamic parameters (Swan & Ganz Combo, Heparin Coated, Edwards Lifesciences, Irvine, CA, USA). The right arteria femoralis was provided with an arterial line for managing hemorrhagic shock (B. Braun Perfusor Line, Melsungen, Germany) and the right vena femoralis with the Icy catheter (ZOLL Circulation, Inc., Sunnyvale, CA, USA) for inducing hypothermia. The urinary output was monitored after preparation of the bladder inserting a suprapubic urinary catheter (Cystofix, B. Braun, Melsungen, Germany).

### 2.4. Polytrauma

Polytrauma was induced at baseline (BL) consisting of blunt chest trauma, penetrating abdominal injury, and hemorrhagic shock. Shock/trauma (S) was maintained over a period of 90 minutes. Resuscitation (R) followed for 60 minutes. Measurements were performed at certain points of time until the end at 15.5 hours (Figure 4). Laboratory and blood gas-parameters hemoglobin (Hb in g/dL), arterial pH, base excess (BE), HCO^3−^, lactate (LC), and glycemia (Gluc) were analyzed (ABL 800 Flex, Drott, Wiener Neudorf, Austria). Respiratory parameters were adjusted if necessary to keep them on a physiologic level at BL. FiO_2_ was reduced from 30% to 21% in sham and in trauma animals just before BL in order to mimic normal physiological and equal conditions at BL. RF was maintained at 22.8 ± 3.0/min in sham and 22.1 ± 3.8/min in trauma animals at BL. At first,* blunt chest trauma* was performed by a bolt shot on a lead panel of 1 cm thickness to the right dorsal, lower chest using cattle killing cartridges (9 × 17, Dynamit Nobel AG, Troisdorf, Germany). The shot was applied while the lungs of the animals were semideflated. Subsequently,* laparotomy* was done by exploration and by two incisions in the right upper liver lobe using a custom-made sharp four-edged scalpel. Then uncontrolled bleeding followed for 30 seconds, afterwards liver packing with 5 sterile packs of the same size. Laceration-associated bleeding was assessed macroscopically every 30 minutes. There were no surgical interventions to stop bleeding after laceration.* Pressure-controlled hemorrhagic shock* was induced by withdrawing blood to a mean arterial blood pressure (MAP) of 30 ± 5 mmHg from the right femoral artery with a maximum of 45% of the total blood volume. Mean blood loss was 880 ± 133 mL in normothermic trauma animals and 829 ± 162 mL in hypothermic trauma animals. Hemorrhagic shock was maintained for 90 minutes. Shed blood was abolished. There were no surgical interventions to stop the bleeding.

### 2.5. Resuscitation

Resuscitation began and lasted for 60 minutes. It followed straight after the period of shock. We approached a mean arterial pressure (MAP in mmHg) of 60 mmHg and a heart rate of less than 150/min, in order to conduct a goal directed therapy only by volume management. Thus, we used colloids (HES 130/4, 6%, Voluven, 308 mosml/L, Fresenius Kabi GmbH, Graz, Austria) and crystalloids (Ringer, 309 mosmol/L, Fresenius Kabi GmbH, Graz, Austria) for fluid resuscitation with four times the shed blood in a relation of 1 : 8 (12.5% hydroxyethyl starch “HES” and 87.5% Ringer) [14, 22]. Sham animals only received crystalloids in a rate of 10 mL/kg bodyweight/hour. Fluid balance was documented. There was no adjunctive medication for resuscitation. Pigs were constantly evaluated and if required treated by current standards of emergency medicine and trauma surgery, that is, Acute Trauma Life Support (ATLS) and European Resuscitation Council (ERC) from 2010 [17, 23]. These protocols allow standard techniques such as inserting chest tubes in case of pneumothoraces and draining blood from cardiac tamponade by needle punctures. Vasopressors, inotropes, and dilators were only applied for life-threatening events such as acute hypotension, ventricular fibrillation, and cardiac arrest.

### 2.6. Normothermia and Hypothermia

Body core temperature (in Celsius °C) was gained continuously by anal wire thermometer (Draeger, Infinity Delta, Danvers, MA, USA). Therapeutic hypothermia (TH) was induced after resuscitation by Icy catheter. A triple infusion lumen was placed in the right vena femoralis and connected to a CoolGard3000/Thermogard XP system (ZOLL Circulation, Inc., Sunnyvale, CA, USA). We targeted a mild hypothermic temperature at 34.3 ± 0.2°C as the porcine physiologic temperature is higher than in humans anyway (38° and 39.5°C) [24, 25]. It took 90 minutes to restore a physiologic temperature of 37°C which was maintained until the end of protocol (Figure 4). Generally, the entire protocol was 15.5 hours long with a short period of 3 hours for TH due to ethical agreements of our model and due to logistical settings at site. We were not allowed to waken the animals afterwards. Therefore we had to shorten the phase of TH within the intensive care phase.

### 2.7. Hemodynamics

Hemodynamic parameters were continuously monitored with noninvasive and invasive methods. A standard electrocardiogram (ECG) monitoring cardiac rhythm, heart rate (HR in 1/min), and invasive mean arterial blood pressure (MAP in mmHg) were deducted. A Swan-Ganz (continuous cardiac output thermodilution/mixed venous oxygen saturation, Edwards Lifesciences, Vigilance, Irvine, CA, USA) was inserted to observe pulmonary hemodynamic pressures, that is, central venous pressure (CVP in mmHg), measurement of mixed venous oxygen saturation (SvO_2_ in %), and cardiac output (CO in l/min). CO and SvO_2_ were displayed on a Vigilance monitor. Additional measurements involved stroke volume (SV in mL), pulmonary artery pressure (PAP systolic, diastolic, and mean in mm Hg), and balloon inflation for wedge pressure (WP in mmHg). PAP was documented 5 min after blunt chest shot. Further total peripheral resistance (TPR in dyn *∗* s *∗* cm^−5^) was documented. Catheter placements and lines were controlled regularly for free air and thrombotic material.

### 2.8. Statistics

The statistical evaluation was carried out with Prism, Version 5.0a (GraphPad Software, Inc., La Jolla, CA, USA). Measurements are presented as means, standard error of the mean with maximum and minimum. The size of each group was *n* = 10. Unpaired *t*-test was calculated when comparing two values between sham and trauma animals. One-way ANOVA and Tukey's multiple comparison tests were performed. The significance was calculated as *P* < 0.05.

## 3. Results

### 3.1. Temperature Control (Figure 1)

**Figure 1:**
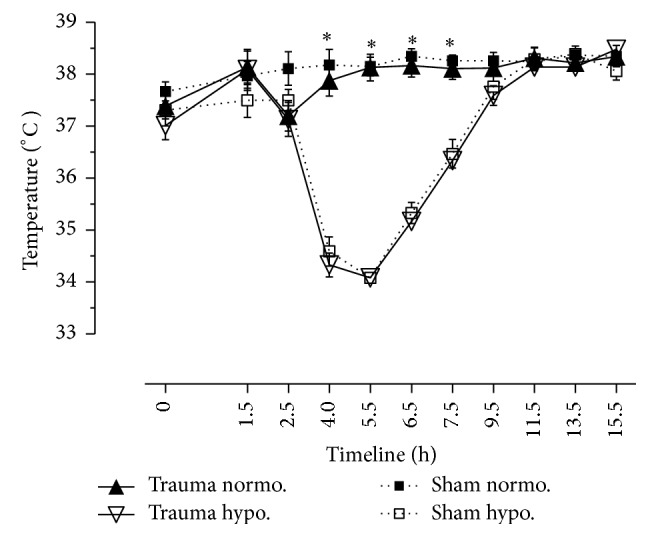
Temperature (in °C). Temperature measured by rectal tube. Normothermic and hypothermic sham animals versus normothermic and hypothermic trauma animals. *N* = 40. Timeline 15.5 hrs. Data are shown in mean and standard error of mean. ^*∗*^ *P* < 0.05, normothermic versus hypothermic pigs during cooling phase (4, 5.5, 6.5, and 7.5 hrs).

Both normothermic and hypothermic sham and trauma animals survived the procedures. Normothermic pigs did not receive endovascular cooling. Both normothermic sham and normothermic trauma animals remained at physiologic body core temperature (Figure 1). Body core temperature of normothermic trauma animals dropped due to dilutionary effects. Although prewarmed fluids were used, temperature decreased from 38.14 ± 0.34°C down to 37.2 ± 0.29°C (*P* < 0.05). There were no significant differences between hypothermic sham and hypothermic trauma animals. We did not observe any arrhythmias due to TH. In hypothermic trauma animals temperature was 37.0 ± 0.3°C at BL; it declined to 34.3 ± 0.2°C at H (*P* < 0.05). Body core temperature was 38.5 ± 0.2°C at the end of protocol (Figure 1).

### 3.2. Hemodynamics (Figures 2(a)–2(j))

**Figure 2:**
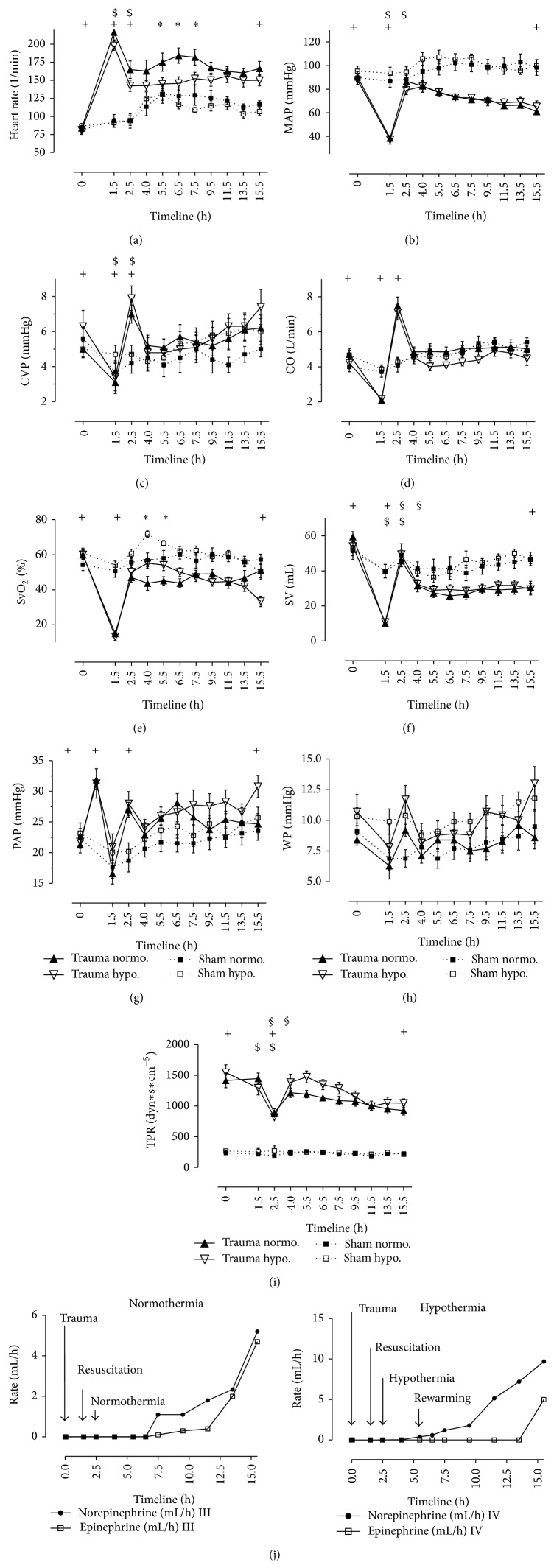
Normothermic and hypothermic sham animals versus normothermic and hypothermic trauma animals. *N* = 40. Timeline 15.5 hrs. Data are shown in mean and standard error of mean. ^+^ *P* < 0.05, values at BL versus values at S and at R and values at E of trauma pigs (0, 1.5, 2.5, and 15.5 hrs). ^*∗*^ *P* < 0.05, normothermic versus hypothermic pigs during cooling phase (4, 5.5 hrs). ^$^ *P* < 0.05, values at S versus values at R in trauma pigs (1.5 and 2.5 hrs). ^§^ *P* < 0.05, values at R versus values at H in trauma pigs (2.5 and 4.0 hrs). (a) Heart rate (HR in 1/min). (b) Mean arterial pressure (MAP in mmHg). (c) Central venous pressure (CVP in mmHg). (d) Cardiac output (CO in l/min). (e) Mixed venous oxygen saturation (SvO_2_ in %). (f) Stroke volume (SV in mL). (g) Pulmonary artery pressure (PAP in mmHg). (h) Wedge pressure (WP in mmHg). (i) TPR (dyn *∗* sec *∗* cm^−5^). (j) Norepinephrine and epinephrine in 3 mg/50 mL (in mL/h).

Hemodynamics occurred as expected after shock (Figures 2(a)–2(i)). HR of normothermic trauma pigs increased to 216.7 ± 11/min (*P* < 0.05), MAP dropped significantly to 38.5 ± 2.7 mmHg (*P* < 0.05), and CVP declined to 3.1 ± 0.5 mmHg (*P* < 0.05) after shock (S). CO declined to 2.1 ± 0.2 l/min and SvO_2_ significantly dropped to 15 ± 2% after polytrauma (*P* < 0.05). PAP reached a significant peak of 31.9 ± 1.6 mmHg 5 minutes due to bolt shot gun (*P* < 0.05). Volume resuscitation led to a significant improvement of these parameters in normothermic and in hypothermic trauma animals. Normothermic trauma animals (*n* = 10) received 3217 ± 259 mL of crystalloids and 445 ± 66 mL of colloids during resuscitation. Hypothermic trauma animals (*n* = 10) received 3295 ± 610 mL of crystalloids and 417 ± 83 mL of colloids during resuscitation. We did not document any fluid loss in the chest. Bleeding, especially rebleeding, was not observed after laceration of the liver. Due to hypothermia, HR was significantly lower than HR in normothermic trauma pigs (Figure 2(a)). After rewarming, HR remained on a stable level and finished with 150 ± 7.7/min. TH had a significant influence on neither MAP nor CVP (Figures 2(b)-2(c)). CO remained on a lower level in hypothermic animals than in normothermic animals (Figure 2(d)). SvO_2_ reached significantly higher values during therapeutic cooling with 34°C (*P* < 0.05). Similar effects were observed in sham animals (Figure 2(e)). The level of SvO_2_ significantly declined after rewarming towards the end of protocol in hypothermic trauma pigs. Values were significantly lower than in sham and in normothermic trauma pigs. TPR showed significant differences among sham and trauma animals throughout the whole protocol. Values already differed at BL between sham and trauma animals. In clinical course, TPR reached four times higher values in hypothermic animals (Figure 2(i)). Vasopressors and inotropes, that is, norepinephrine and epinephrine, were used only in a very few animals in this trauma model. Sham animals did not need catecholamine medication. Five normothermic trauma animals and one hypothermic trauma animal needed catecholamine medication. This medication was started after rewarming in all cases. Dosages were applied with either epinephrine or norepinephrine in 3 mg/50 mL (Figure 2(j)).

### 3.3. Laboratory Parameters and Blood Gases (Figures 3(a)–3(c))

**Figure 3:**
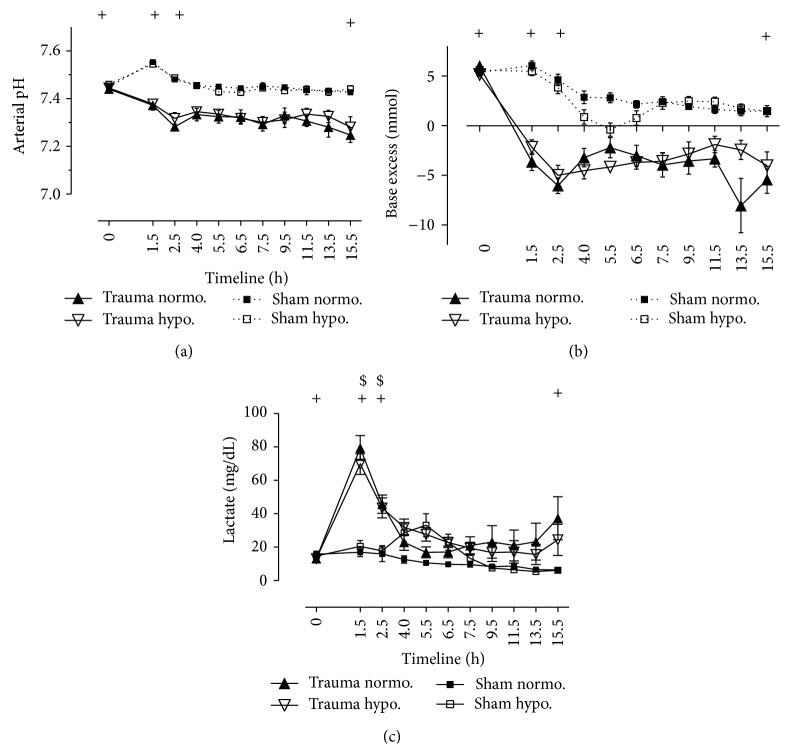
Blood gases and respiratory parameters. Normothermic and hypothermic sham animals versus normothermic and hypothermic trauma animals. *N* = 40. Timeline 15.5 hrs. Data are shown in mean and standard error of mean. ^+^ *P* < 0.05, values at BL versus values at S and at R and values at E of trauma pigs (0, 1.5, 2.5, and 15.5 hrs). ^$^ *P* < 0.05, values at S versus values at R in trauma pigs (1.5 and 2.5 hrs). ^§^ *P* < 0.05, values at R versus values at H in trauma pigs (2.5 and 4.0 hrs). (a) Arterial pH. (b) Base excess (BE in mmol/L). (c) Lactate in mg/dL.

**Figure 4:**
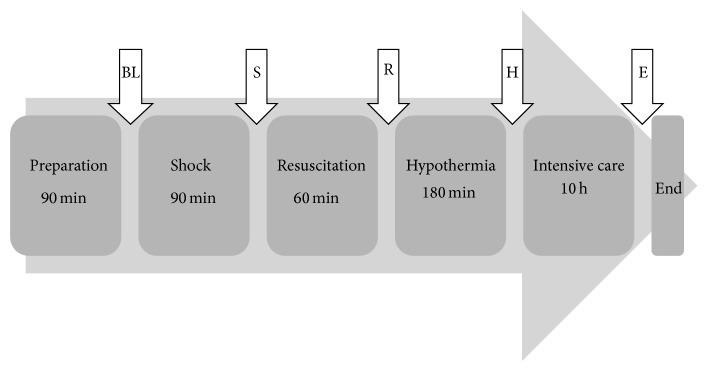
Timeline. The timeline of our porcine polytrauma model is displayed. The measurements were performed at several points of time during protocol: at baseline (BL) straight after 90 minutes of preparation, after 90 minutes of shock (S), after 60 minutes of resuscitation (R), after 180 minutes of therapeutic hypothermia (H), and at the end of protocol (E) (15.5 hours after BL).

Our polytrauma model with blunt chest trauma, penetrating abdominal trauma, and hemorrhagic shock caused a depletion of laboratory parameters and blood gases (Figures 3(a)–3(c)). Hemoglobin (Hb in g/dL) decreased from 9.5 ± 0.3 g/dL in normothermic trauma animals and from 9.1 ± 0.4 g/dL in hypothermic trauma animals at BL down to 5.2 ± 0.2 g/dL at R. The depth of shock was displayed through decreased base excess (BE) at −3.6 ± 0.9 mmol/L and increased lactate (LC) at 79.4 ± 7.4 mg/dL evaluated after trauma [14]. Physiologic arterial pH is between 7.39 and 7.45 in porcine model [26]. Both sham and trauma pigs in our study had an arterial pH of 7.44 at BL. It continuously decreased down to 7.25 ± 0.03 in normothermic trauma pigs and to 7.28 ± 0.04 in hypothermic trauma animals at E (*P* < 0.05). There were no significant differences due to temperatures. Base excess significantly dropped to −6 ± 0.8 mmol/L in normothermic trauma animals and down to −5 ± 0.9 mmol/L in hypothermic trauma animals. In sham pigs hypothermia showed more negative base excess than in normothermia. TH produced higher levels of lactate than normothermia at point of time 5.5 hrs (*P* < 0.05). Glycemia undulated within a physiologic range of glucose (in mg/dL) between 70 and 120 mg/dL. Sham pigs showed physiologic values in urinary output. Urinary output started with 214 ± 58 mL/h in normothermic trauma animals and with 160 ± 46 mL/h in hypothermic trauma animals at BL. The urinary output decreased to values of 69 ± 23 mL/h and 40 ± 8 mL/h, respectively, after shock. There were no significant differences among hypothermic trauma animals and normothermic trauma animals.

## 4. Discussion

### 4.1. General

Up to date, thoracic and abdominal trauma with hemorrhagic shock are hazardous risks leading to sepsis and consecutively multiorgan failure (MOF) [27]. Mortality still displays high rates. Therefore, we have previously developed a porcine polytrauma model with settings under realistic conditions [14].

But, do any new therapy strategies exist for intensive care medicine? Thus, we chose to establish a mild therapeutic hypothermia* after* polytrauma and resuscitation. Our timeline for TH (3 hours) was short as already mentioned above. Due to past examinations on therapeutic hypothermia in animal studies the evidence of the optimal length, duration, and depth of TH is still unanswered [28]. Furthermore as our protocol ended at 15.5 hrs it was not possible to observe clinical outcome after wakening of the animals. But this goal was subsidiary because the acute course of hemodynamics due to TH was of higher interest. The simulated phase of intensive care displayed a very vulnerable phase for the polytrauma patient as chosen for this study [29, 30]. Most of all we aimed to avoid any interfering effects of TH during hemorrhagic shock [31]. Unfortunately therapeutic hypothermia has rarely been described as a therapy option after polytrauma. Nevertheless the need for a new approach is reasonably high. Clinical and experimental studies have highlighted the delirious impact of accidental hypothermia (AH) on the patients' clinical course [32–36]. On the other side the therapeutic approach of hypothermia got in the focus of public interest as recommendations of the International Liaison Committee on Resuscitation had been published dated back in 2002 [1]. Surgical facilities have been performing therapeutic hypothermia (TH) for many years using its tissue protective effects [3, 37]. Unfortunately full knowledge on beneficial and harmful impact of hypothermia is still lacking. Improved neurological outcome is described due to TH after cardiac arrest [5, 38, 39]. Current literature demonstrates almost equal neurological outcome of unconscious survivors of out-of-hospital cardiac arrest who were treated by either hypo- or normothermia [6]. TH even emerges as a therapeutic approach for traumatic brain injury (TBI) [4, 5]. In patients with TBI the superior effect is still to be proven because in previous clinical studies groups were small and rather not representative [7]. Furthermore active cooling that is in polytrauma is still under experimental development. Previous investigations on porcine model have shown that environmental hypothermia in hemorrhage and polytrauma has no significant impact on organic and neurological function [40]. Therefore it is of uttermost interest to extend studies concerning therapeutic hypothermia in polytrauma care.

### 4.2. Temperature

Due to past experiments temperature of TH is suggested to be between 32 and 34°C [41]. Therefore we chose a body core temperature of 34°C to follow those recommendations. It has to be considered that the porcine physiologic temperature is higher than in humans (38° and 39.5°C) [24, 25]. We gained the body core temperature by rectal tube which was connected to CoolGard3000/Thermogard XP as technically required. Other studies preferred temperature measurements by tympanic membrane, bladder, and esophageal or pulmonary catheter. Temperature differences did not vary significantly between rectal and pulmonary artery catheter samples in our study. Furthermore, volume management displays an important role as it can induce AH [36]. Prewarmed fluids are generally suggested [23]. Although resuscitation fluids were prewarmed prior to reperfusion in our study, temperature slightly dropped in trauma animals due to resuscitation. TH can be performed in several ways. Yet, no standard method has been succeeded as noninvasive and invasive cooling devices have to be differentiated. We chose an invasive method for cooling (Icy catheter, CoolGard3000/Thermogard XP system). This catheter is inserted percutaneously with an inner circulating water system driven by an external device. It is working safely at a constant rate of cooling. Both in hypothermic sham and in hypothermic trauma animals temperature was reduced down to 34.3 ± 0.2°C. Shivering was observed. Rewarming increased body core temperature to physiological levels. Animals remained stable during this period. Sunde et al. reported on similar experiences with endovascular cooling devices [42]. But the extended intraluminal placement of catheters might facilitate inflammatory risks and complications. Other cooling techniques include the infusion of IV cold fluids, which are used in preclinical settings after cardiac arrest and ROSC [43]. Another approach is an extracorporeal system with rapid cooling rates, further supplying oxygen and drugs [44, 45]. More invasive possibilities are cold peritoneal lavages, intraventricular cerebral hypothermia, and nasal and gastric lavages [46, 47]. Other studies have used external applications for temperature management, that is, cooling blankets, ice packs, icy fluids, alcohol, and fans [31, 48], which are simple in use but insufficient for targeting temperature [49].

### 4.3. Hemodynamics

Porcine hemodynamics is comparable to the human system [50]. Several groups have worked on TH during and after hemorrhagic shock [32, 51–56]. Still, the impact of TH after severe trauma is unknown. We aimed to unfold both beneficial and harmful impacts of TH. Concerning monitoring tools invasive devices such as Swan-Ganz are controversially discussed because of potential surgical bleedings and because of maladapted placements [57, 58]. We did not observe any complications. Impact of shock was reflected by an increase of HR and decrease of MAP [14] (Figures 2(a)-2(b)). Values of CO and SV dropped significantly after shock. Arterial bloodline and Swan-Ganz balloon catheter continuously documented cardiovascular parameters as early goal-directed therapy decreases morbidity and mortality [16]. Thus, cardiovascular parameters, mainly HR and MAP, improved as mentioned above due to fluid management with colloids and crystalloids in a relation of 12.5% HES and 87.5% Ringer according to current trauma guidelines [3, 22]. Colloids lose their relevance in emergency care, although these components expand plasma volume up to 80% [1, 4]. Current discussions (VISEP, 6S, and CHEST) dispute the use of colloids in critically ill patients and the jury is still out as colloids indeed were used in the initial stabilization phases in these studies [59, 60]. Due to technical reasons at site we had to abolish shed blood. Nevertheless, cardiovascular parameters adequately improved without the general use of vasopressors or inotropes [61]. Reperfusion led to physiologic values. It is well known that rapid restoration of hemodynamics improves the tissue oxygenation as well as cellular functions [28, 62, 63]. Further to investigate on TH, we accede to past studies that TH shields cardiac function [31]. Past investigations described increased oxygen concentrations in systemic circulation after TH and hemorrhagic shock [64]. Mild hypothermia has been attributed to show decreased heart rate and decreased cardiac output but an increased vascular resistance. Mean arterial blood pressure and stroke volume are maintained under TH [41]. We share these findings as HR significantly and CO nonsignificantly decreased in hypothermic trauma animals in comparison to normothermic trauma animals (Figures 2(a) and 2(d)). Most interestingly, mixed venous oxygen saturation was significantly higher in hypothermic trauma animals than in normothermic trauma animals as shown in our experimental approach (Figure 2(e)). But unfortunately SvO_2_ significantly decreased after rewarming and reached lower values in hypothermic than in normothermic trauma pigs. The mechanism of effective oxygen delivery during TH is explained by reduced metabolic rates; ATP is preserved [65]. Hypothermia reduces apoptosis and necrosis [8]. Moreover the depth of moderate temperature (34°C) determines the decrease of myocardial needs while cardiovascular function is stable in our model [31]. Nevertheless lactate values were high in both normo- and hypothermic trauma animals at the end of protocol, which might explain that hypothermic conditions have no positive effect on metabolism at all, at least under this short period of time. The fact of positive inotrope effect due to hypothermia is supported by our findings concerning the stroke volume (SV) which stayed on a lower level in normothermic and hypothermic trauma pigs than in normothermic and hypothermic sham pigs (Figure 2(f)). Our chosen moderate hypothermia provides no increased inotropic effects supported by Meyer and Horton [31]. The systemic vascular resistance (TPR) significantly increased during TH in relation to normothermic trauma animals. But, interestingly, both normo- and hypothermic sham animals generally stayed on a lower level than trauma animals. There were no differences among normothermic and hypothermic sham animals (Figure 2(i)). We can just assume that the preparation phase might have influenced TPR in its results but this is in contrast to other cardiac parameters, which remain unaffected. After rewarming, values of SvO_2_ and MAP declined (Figures 2(b) and 2(e)). In addition, the results of blood gases due to hypothermia are of no surprise. Values have to be carefully analyzed as the solubility of blood gases increases when temperature decreases [41].

Another approach of this study which is already published by Mohr et al. [20] was the observation of coagulopathy in porcine model. Unfavorable impact of hypothermia affects the platelet function and harmfully prolongs prothrombin and partial thromboplastin times [66]. Furthermore, we did not observe macroscopically rebleeding potential in our porcine model. Studies on rodent animals have shown that there is a risk of rebleeding due to hypothermia [34]. It has to be known that these findings were conducted under deeper cooling at 30°C. Generally swine are frequently used in trauma models and they show high stability concerning coagulation pathophysiology [67]. Another fact on coagulopathy due to hemorrhage could be taken from clinical studies on patients who sustained trauma incidences; coagulopathy was triggered by several pathways, that is, activation of protein C, malfunction of endothelial borders, platelet dysfunction, and primary and secondary fibrinolysis because of tissue injury. Concepts like damage control resuscitation (DCR) work which includes the transfusion of ratios of fresh frozen plasma (FFP) and red blood cells (RBC) [68, 69].

## 5. Conclusions

Our findings suggest that TH can be safely performed at 34°C in a porcine model. TH was not detrimental for cardiac function in porcine model. Hemodynamics was rather stable during TH. Interestingly, TH provides hints for tissue protective impact. Values of SvO_2_ were high during TH. But, presumably, length and depth of TH were not intensive enough to clearly substantiate the advantages and disadvantages of TH. Further studies should extend the duration of TH.

## Abbreviations

ATLS: Acute Trauma Life Support
ATP: Adenosine triphosphate
AH: Accidental hypothermia
BE: Base excess, in mmol/L
BL: Baseline, starting point, 0 minutes
CO: Cardiac output, in L/min
CO_2_: Carbon dioxide
CVP: Central venous pressure, in mmHg
ECG: Electrocardiogram
et CO_2_: Endexspirational carbon dioxide, in %
FiO_2_: Fraction of inspirational oxygen, in %
H: Point of measurement after hypothermia, 180 minutes after R
HES: Hydroxyethyl starch, 12,5%
HR: Heart rate, in 1/min
HCO_3_^−^: Hydrogen carbonate
IV: Intravenous
LC: Lactate, in mmol/L
MAP: Mean arterial blood pressure, in mmHg
MOF: Multiorgan failure
PAP: Pulmonary artery pressure, in mmHg
PaCO_2_: Partial pressure of carbon dioxide
PaO_2_: Partial pressure of oxygen
PEEP: Positive endexspiratory pressure, in mmHg
ROSC: Return of spontaneous circulation
R: Point of measurement after resuscitation, 60 minutes after S
RF: Respiratory frequency
S: Point of measurement after shock, 60 minutes after BL
SvO_2_: Mixed venous oxygen saturation, in percent (%)
SpO_2_: Peripheral oxygen saturation, in percent (%)
SV: Stroke volume, in mL
TBI: Traumatic brain injury
TH: Therapeutic hypothermia, here defined as temperature in 34° Celsius
TPR: Total peripheral concentration
Vt: Tidal volume, in mL
WP: Wedge pressure, in mmHg.
